# The liver's unexpected ally: on possible liver protection against metastases by hydatid cysts

**DOI:** 10.17179/excli2025-8635

**Published:** 2025-08-08

**Authors:** Bachir Benarba, Noureddine Bachir Bouiadjra, Atanasio Pandiella

**Affiliations:** 1 Laboratory Research on Biological Systems and Geomatics, Mustapha Stambouli University of Mascara, Algeria; 2 Thematic Research Agency in Health and Life Sciences, Oran, Algeria; 3 Instituto de Biología Molecular y Celular del Cáncer and CIBERONC, CSIC-Universidad de Salamanca, Campus Miguel de Unamuno, Salamanca, Spain

## ⁯⁯⁯⁯

During more than 40 years as surgeons (NBB), we have continuously observed a surprising clinical pattern: cancer patients with hydatic cysts did not develop liver metastases from distant primary cancers. These persistent clinical observations led us to hypothesize that the hydatic cysts or its causal agent may expert a protective effect against metastatic liver cancer. 

It seems that several factors generated by the hydatic cyst or the parasite, such as the physical barrier or immune modulation, could be responsible for this possible protective effect. By investigating these mechanisms, researchers could understand how to prevent liver metastases. 

### Mechanical barrier 

The fluid-filled cysts following the parasite-induced lesions lead to an inflammatory response that results in the creation of a dense extracellular matrix (ECM) protective layer over the lesions (Hamad et al., 2024[6]). This barrier, called the pericyst, is made of collagen produced by activating fibroblasts, fibroblasts, and immune infiltrates, and may serve as a mechanical and biochemical barrier against the invasion of metastatic cells and evasion of the parasite. Invasion of the liver tissue and movements of cancer cells may be impeded and prevented by the dense extracellular matrix (Lu et al., 2012[8]).

Although a stiff extracellular matrix has been linked to drug resistance and tumor progression in certain cancers, such as pancreatic cancer (Piersma et al., 2020[11]), it can change cellular behavior and impede the epithelial-to-mesenchymal transition associated with the development of metastases. 

### Immune response

Although the Th1-type immune response has been reported to be associated with the infection in intermediate hosts, the Th2-type response remains the immune response in humans and animals with involvement of different cytokines such as IL-4, IL-5, IL-10, and IL-13 and inflammatory cells including mast cells, lymphocytes, eosinophils, and macrophages (De Biase et al., 2023[3]). While the pro-inflammatory response was associated with the Th1 type immune response, the Th2 type was characterized by a protective immunoregulatory effect with significant levels of IL-4, IL-10 and TGF-β (Privitera et al., 2024[12]). These anti-inflammatory cytokines improve mitochondrial respiration, oxidative metabolism and regulatory T-cell activity. The hydatid cyst-induced Th-2 immune response may create a disadvantageous environment for metastatic cancer cells. Most recently, Jacenik et al. (2025[7]) showed that the Th-2 immune response reprograms the tumor microenvironment through macrophage polarization, affecting the activation of both macrophages and eosinophils, which then results in the massive release of cytotoxic and apoptotic molecules and inhibits colon and pancreas tumor growth. Thus, it has been suggested that the Th2 response could become a promising target once it has been associated with enhanced antitumor immunity (Jacenik et al., 2023[7]). In the tumor microenvironment, activated eosinophils recruit CD8+ T cells through their chemokines (CCL-5, -9 and -10), thus giving rise to a polarization of macrophages toward the M1-like antitumor phenotype (Carretero et al., 2015[1]). Likewise, eosinophils recruited and activated natural killer cells into the tumor microenvironment, thereby enhancing the anti-tumor immune reaction (O'Flaherty et al., 2017[10]). 

On the other hand, the inhibition of lymphangiogenesis resulting from the Th-2 immune response may contribute to the anti-metastatic and anti-angiogenic effects. Indeed, Savetsky et al. (2015[14]) found that Th-2 cytokines exerted an important anti-lymphangiogenic (in vitro and in vivo) through the induction of apoptosis of lymphatic endothelial cells, inhibition of their migration and tubule formation, increasing fibrosis and significant changes in the extracellular matrix. Furthermore, they found that the Th-2 immune response (IL-4 and IL-13) significantly downregulated the expression of Prox-1, considered as a master regulator of lymphangiogenesis. 

On the other hand, eosinophils activated by Th-2 cytokines, can contribute to this anti-tumor effect by infiltrating tumors and inhibiting their growth (Ellyard et al., 2007[4]). Activated eosinophils were demonstrated to have direct contact with cancer cells and exert an anticancer effect against them, even in the absence of NK or T cells, through the release of cytotoxic agents such as ROS, granzyme A, granzyme B, TNF-α, MBP (Major Basic Protein), and ECP (Eosinophil Cationic Protein). These molecules were shown to infiltrate tumors, including liver metastases, thereby exerting anti-tumor effect (Ghaffari and Rezaei, 2023[5]). In a recent study, Motavallihaghi et al. (2023[9]) found that the crude hydatid cyst fluid inhibited colon cancer cells through the induction of apoptosis and inhibition of anti-apoptotic genes. 

### Cross-reactive immunity

Another mechanism involves cross-reactive immunity. Indeed, it has been reported that the frequent presence of microbe-specific T cells in the tumoral microenvironment results in improved immune surveillance by exploiting pre-existing effector mechanisms against nascent malignant tumors (Zitvogel and Kroemer, 2022[16]). Moreover, it has been demonstrated that microorganisms can prime T cells to recognize tumor-associated antigens (TAAs), and possibly control tumor growth (Cavalluzzo et al., 2024[2]). Here we hypothesize that a chronic infection with *Echinococcus*, and thereby through EC antigens EG95 and Eg31, may prime both T cells and macrophages to recognize TAAs and therefore eliminate cancer cells. Actually, owing to their potential to induce a strong immune response, EC antigens (especially in the protoscolex) have been studied for developing a possible cancer immunotherapy (Safahi and Ahmadi, 2024[13]). Moreover, chronic EC infection is associated with a continuous low-level antigen leakage into the liver tissue, which in turn leads to activation of Toll-like receptors (TLRs) and PRRs expressed by the liver tissue (dendritic cells, Kupffer cells, sinusoidal endothelial cells) (Vázquez-Mendoza et al., 2013[15]). The persistent activation of these receptors results in an “immune alertness” or a state of “trained anticancer immunity” that could prevent liver metastases. Therefore, further studies are needed to investigate this promising path about a “EC-infection-trained” anti-tumor microenvironment within the liver. 

In spite of the reported long-term clinical observations, important factors should be considered such as the patient immune status, cancer type, and the nature of hydatic cyst for future systematic and mechanistic studies to demonstrate whether an effective protective effect of hydatic cyst infection against liver metastases exists, and if so, to clarify the different mechanisms involved. Notably, future research should focus on the molecular and immunological mechanisms occurring within the hydatic-infected liver tissue microenvironment, to confirm a possible induced “immune alertness” or therapeutic potential of parasite-derived molecules leading to the prevention of liver metastases. 

If this hypothesis is experimentally and epidemiologically validated, it could open the way to innovative cancer prevention strategies or immune therapies that can be used especially for the prevention of metastases in patients with high-risk cancer.

In conclusion, our long-standing clinical observations suggest that hepatic hydatid cysts may confer an unexpected protective effect against liver metastases. Our hypothesis suggests several mechanisms responsible for this effect, including the mechanical barrier mechanism formed by the pericyst, immunomodulation within the hepatic tissue, and cross-reactive immunity leading to immune alertness. By exploring these different pathways, future research could develop novel preventive or therapeutic strategies against liver metastases.

## Conflict of interest

The authors declare no conflict of interest.
